# When mission meets isolation: a five-wave longitudinal study of how workplace loneliness erodes job embeddedness through role stress and organizational identification among railway police

**DOI:** 10.3389/fpsyg.2026.1891131

**Published:** 2026-07-29

**Authors:** Bingquan Chen, Songze Zhu, Shi Jin, Yifei Hou, Tianyu Zhang

**Affiliations:** 1 School of Public Security Administration, People’s Public Security University of China, Beijing, China; 2 School of Information Network Security, People’s Public Security University of China, Beijing, China

**Keywords:** job embeddedness, organizational identification, public service motivation, railway police, role stress, workplace loneliness

## Abstract

**Introduction:**

Workplace loneliness is increasingly recognized as a critical concern in occupational health psychology. However, the sequential mechanisms through which it translates into organizational detachment remain poorly understood, particularly in high-loneliness occupations such as railway policing.

**Objectives:**

Drawing on Social Identity Theory and Equity Theory, this study proposed and tested a moderated serial mediation model. The model examined two research questions: (a) whether workplace loneliness predicts lower job embeddedness through the serial mediation of role stress and organizational identification, and (b) whether public service motivation (PSM) moderates this process.

**Methods:**

A five-wave longitudinal design with one-month intervals was employed. Data were collected from 736 active-duty railway police officers in China, a population characterized by prolonged solitary patrols and fragmented peer interaction. Workplace loneliness (T1), role stress (T2), organizational identification (T3), PSM (T4), and job embeddedness (T5) were measured across five time points.

**Results:**

Results showed that workplace loneliness indirectly predicted lower job embeddedness through the serial mediation of role stress and organizational identification. The total indirect effect accounted for 59.1% of the total effect. Notably, PSM amplified rather than buffered the negative association between workplace loneliness and job embeddedness. Specifically, this negative association was stronger among officers with higher levels of PSM.

**Discussion:**

These findings advance the workplace loneliness literature in two ways. First, they provide longitudinal evidence for a sequential erosion pathway—from role cognition disruption to identity weakening to organizational detachment. Second, they reveal a paradoxical dark side of PSM in resource-depleted organizational contexts. The results also carry practical implications for designing targeted psychological interventions and organizational support systems for isolated occupational groups. More broadly, they highlight the value of longitudinal designs in public safety populations.

## 1 Introduction

The railway system is evolving rapidly. As high-speed rail networks continue to expand, a shortage of railway police officers has become increasingly acute (Wang, 2025). The single-officer patrol model, now firmly entrenched, has led to a lack of teamwork. Officers are dispersed across different worksites and rotate through irregular shifts, which further fragments peer interaction. These conditions pose serious threats to railway police officers’ psychological belonging and career stability.

Concepts such as workplace loneliness (WL), job embeddedness (JE), role stress (RS), organizational identification (OI), and public service motivation (PSM) have been widely applied in organizational behavior and occupational health psychology research. However, railway police officers work as “lone fighters”—they endure prolonged isolation, enforce the law in confined train compartments, and lack peer reference. These occupational features make the mechanisms through which the above psychological constructs operate all the more complex. Notably, as Chung et al. (2022) observed, research on WL among police officers remains in its early stages, and its proximal and distal effects on work attitudes and behaviors have yet to be systematically investigated. Therefore, investigating how these constructs interact in the context of railway policing will help illuminate officers’ occupational behavioral motivations and psychological detachment processes, thereby providing both theoretical and practical support for optimizing psychological intervention strategies and strengthening their organizational attachment.

## 2 Literature review

Despite growing scholarly attention to WL and JE, our understanding of how the former translates into the latter remains limited. We tentatively identify three potential gaps that the present study seeks to explore.

First, the sequential psychological mechanisms linking WL to JE remain largely unexplored. Existing research on the antecedents of JE has examined organizational socialization tactics (Allen, 2006), organizational culture (Ramesh and Gelfand, 2010), and leadership (Hom et al., 2009), yet how individual psychological perceptions connect WL to JE has received limited attention. Most studies have focused on the direct effects of WL on turnover intention or work attitudes in general organizational settings (Ertosun and Erdil, 2012; Lam and Lau, 2012), leaving intermediate processes uncharted. For railway police—who work in enclosed environments with fragmented interactions and prolonged solitary patrols—the mechanisms through which WL transmits to JE remain insufficiently explained through the lens of social psychological theory. In particular, the potential chain from role cognition disruption to identity weakening has not been systematically examined in high-loneliness occupational contexts.

Second, the temporal ordering of these relationships remains methodologically underdetermined. Although prior research has confirmed the negative predictive effect of RS on OI (Bright, 2022; Wu, 2019), most existing studies have relied on cross-sectional designs, which cannot rule out reverse causality or provide robust evidence for the directionality of effects (Maxwell et al., 2011). The erosion of OI may be a cumulative process rather than a static event: WL may expose individuals to prolonged ambiguous self-categorization, during which RS gradually accumulates and OI is progressively eroded. This suggests that the impact of WL on JE may operate along a serial transmission chain—a possibility that requires longitudinal data to verify.

Third, the boundary conditions of this process—particularly the role of PSM—are insufficiently understood. Although PSM is typically conceptualized as a personal resource that buffers job demands, emerging evidence suggests it may have a “dark side” in resource-depleted contexts (Schott and Ritz, 2018). Whether PSM serves as a protective factor or, paradoxically, an amplifier of loneliness-induced detachment among railway police officers has yet to be empirically tested.

Taken together, these gaps suggest that further investigation is needed to understand how and under what conditions WL erodes JE. Drawing on Social Identity Theory and Equity Theory, the present study represents a preliminary attempt to address these gaps by proposing a moderated serial mediation model tested with a five-wave longitudinal design among Chinese railway police officers.

### 2.1 Social identity theory

Social identity theory, proposed by Tajfel and Turner (1979), centers on the idea that individuals derive part of their self-image from the social groups to which they belong, along with the value and emotional meaning attached to that membership. According to the theory, people build their social identity through three basic psychological processes—social categorization, social comparison, and positive distinctiveness—driven by the fundamental need to maintain and enhance self-esteem. When a positive social identity cannot be attained, individuals may adopt strategies such as social mobility, social creativity, or social competition.

Turner (1985) extended this framework by proposing self-categorization theory, which emphasizes the process of depersonalization. Through depersonalization, individuals come to see themselves less as unique persons and more as embodiments of the ingroup prototype, thereby completing the shift from “I” to “we”.

The single-officer patrol model that railway police officers have long endured systematically severs the social comparison and positive distinctiveness processes essential for social identity construction. As Tajfel (1982) noted, intergroup comparison is a key means through which group members achieve a positive identity: individuals tend to assign positive traits to the ingroup and negative traits to the outgroup, thereby enhancing their self-evaluation. Yet in their isolated law enforcement environment, railway police officers lack real-time interaction with ingroup members—their colleagues. They are unable to confirm the appropriateness of their actions through shared understanding of “our way of doing things.” This deprivation of social comparison information creates an “identity vacuum.” This finding aligns with the central result of the present study—that WL (T1) significantly predicts the decline of JE (T5)—and essentially captures the cognitive precursor of individuals initiating social mobility strategies when positive social identity is out of reach.

### 2.2 Equity theory

Equity theory, proposed by Adams (1965), holds that individuals are concerned not only with the absolute amount of rewards they receive but also with the relative amount—that is, the ratio of their own inputs to outcomes compared against a referent other. Specifically, people judge fairness by comparing their input-to-reward ratio with that of a referent, such as a colleague, their expected self, or an industry standard. When the two ratios are roughly equal, a sense of equity and inner calm prevails. When one’s own ratio falls below that of the referent, dissatisfaction arises, accompanied by feelings of being shortchanged or that things are worse than before. When one’s own ratio exceeds that of the referent, guilt may emerge from having benefited disproportionately. Importantly, both fairness and unfairness are subjective perceptions that depend largely on an individual’s cognitive appraisal, value system, and behavioral norms (Sun and Huang, 2004).

Railway police officers with high PSM carry a strong sense of mission to serve the public and uphold justice. This sense of mission constitutes a core element of their personal input. WL, however, can be seen as a form of organizational return—and a meager one at that. For highly mission-driven officers, it creates a profound imbalance between what they give and what they receive. When confronted with a strong sense of inequity, Adams (1965) noted that individuals may adopt one of five responses: rationalizing the situation to create an illusion of fairness, attempting to alter the other party’s inputs or outcomes, adjusting their own inputs or outcomes (e.g., reducing effort as a form of passive resistance), switching to a different comparison referent, or simply walking away from the job. Thus, paradoxically, in a lonely workplace context, officers with higher PSM may experience greater frustration and a stronger inclination toward psychological withdrawal precisely because of the mismatch between their deep commitment and the meager relational rewards they receive.

### 2.3 WL and JE

WL refers to the distressing experience that arises when there is a gap between the quantity and quality of interpersonal relationships an employee expects to have at work and what they actually obtain, and the employee feels unable to change this imbalance (Wright, 2005). JE is defined as the combined forces that keep an individual from leaving a job (Mitchell et al., 2001). It encompasses three elements—links, fit, and sacrifice—and captures the overall degree of attachment between an individual and their work role. It is a key predictor of employees’ intention to stay and their job stability (Liao et al., 2023).

These two constructs are closely intertwined. Research has shown that the single-officer patrol model intensifies railway police officers’ WL within the confined space of a train. This loneliness can erode their collective identity and sense of belonging, which in turn reduces their work motivation and undermines their JE (Shanghai Railway Public Security Bureau Office Research Group, 2022). At the same time, WL has been linked to elevated burnout (Bryan et al., 2023), diminished work engagement and productivity (Galanti et al., 2021), and heightened negative emotions and psychological distress (Günther et al., 2022)—all of which can further weaken an individual’s perceived links, fit, and sacrifice with the organization. Moreover, the negative impact of WL on organizational commitment and turnover intention has shown cross-contextual stability (Ertosun and Erdil, 2012; Lam and Lau, 2012). As a multidimensional construct encompassing links, fit, and sacrifice, JE captures these negative effects in a more comprehensive manner.

However, most existing studies have focused on the direct effects of WL on turnover intention or work attitudes in general organizational settings. Social identity theory holds that individuals build their social identity and gain positive self-evaluation and self-esteem through three basic processes: social categorization, social comparison, and positive distinctiveness. When a positive social identity cannot be attained, individuals may adopt strategies such as social mobility, social creativity, or social competition to restore a positive self-concept (Yan, 2016). The railway police profession, characterized by high risk, high stress, and the single-officer patrol model, creates a social identity construction environment that differs markedly from that of ordinary organizations. When the group interaction essential for social comparison and positive distinctiveness is persistently blocked, the transmission pathway from WL to JE may exhibit more complex features. Drawing on the core logic of “identity threat—social mobility” in social identity theory, the following hypothesis is proposed:

*H1*: WL as perceived by railway police officers significantly and negatively predicts their JE.

### 2.4 RS and OI

A core contribution of social identity theory lies in revealing how group membership provides individuals with the emotional and value foundation for self-definition (Tajfel and Turner, 1979). According to the theory, the self-concept is built through three interconnected psychological processes—social categorization, social comparison, and positive distinctiveness. Individuals first place themselves into a social category, then affirm the positive distinctiveness of their ingroup through comparison with outgroups, and ultimately achieve enhanced self-esteem and a stable self-concept (Tajfel, 1982; Yan, 2016). Self-categorization theory, developed from this foundation, emphasizes that depersonalization is the key mechanism for achieving group identity. Through depersonalization, individuals come to see themselves as embodiments of the ingroup prototype rather than as unique persons, thereby completing the shift from “I” to “we” (Turner, 2010). For railway police officers, this identity construction process depends heavily on daily interactions with colleagues. By observing how peers handle complex situations and exercise discretion in law enforcement, officers continuously internalize group norms and develop clear role perceptions and a solid sense of organizational belonging. The single-officer patrol model, however, systematically blocks this process, creating conditions in which RS can develop and OI can erode.

First, WL intensifies RS. RS refers to the psychological burden produced by ambiguity, conflict, and overload in work role expectations (Kahn et al., 1964). Viewed through the lens of self-categorization theory, RS can be understood as a cognitive dilemma of self-categorization: when individuals cannot firmly place themselves into a social category with clear norms, role ambiguity, role conflict, and role overload emerge in succession. Turner (1985) argued that the core of self-categorization lies in individuals defining themselves according to the ingroup prototype, and acquiring this prototype depends on information exchange and behavioral referencing in social interaction. However, railway police officers who work for extended periods without colleague interaction lose the basic conditions for observing and learning the group prototype. When WL severs this referencing system, individuals struggle to identify the group‘s behavioral norms in specific situations—this constitutes the cognitive root of role ambiguity. Meanwhile, a lack of social support in the workplace significantly heightens individuals’ perceptions of RS (Chiu et al., 2017). In high-pressure situations such as emergency response, railway police officers often must single-handedly undertake tasks that would normally be handled by a team. The sharp contradiction between duty demands and individual capacity breeds role conflict and role overload. RS, in turn, severs the identity-based bonds that tie individuals to their organization, thereby undermining JE. From the perspective of identity theory, individuals derive a sense of self from their occupational and organizational roles, and they are motivated to verify these role identities through ongoing social interaction and feedback (Stets and Burke, 2000). RS—particularly role ambiguity and role conflict—disrupts this verification process: when individuals cannot clearly perceive what is expected of them or find themselves torn among incompatible demands, they experience a weak verification of their role identity, which generates negative emotions and psychological discomfort that weaken their willingness to remain in the organization. In the context of JE, the psychological bonds that keep an individual attached to their job—links, fit, and sacrifice—are fundamentally identity-based: employees stay not only because they have social connections and perceive a good fit, but because they derive a meaningful sense of who they are from their organizational membership. When RS undermines the clarity and coherence of one‘s role identity, this identity-based foundation of embeddedness erodes (Qiang and He, 2023). For railway police officers, whose professional identity is central to their self-concept and whose role expectations are particularly salient, the damaging effect of RS on organizational attachment may be especially pronounced. Accordingly, the following hypothesis is proposed:

*H2*: RS mediates the relationship between WL and JE.

Second, WL also impairs JE by weakening OI. OI refers to an individual’s perception of oneness with or belongingness to an organization, a sense of identification that aligns the individual’s behavior with the organization’s goals and values (Mael and Ashforth, 1992). Ashforth and Mael (1989), after bringing social identity theory into the organizational context, pointed out that the formation of OI is a concrete manifestation of the social identity process at the organizational level—individuals gain a sense of “who we are” by integrating organizational membership into their self-concept. WL severs the social interaction foundation that this integration process requires. Studies have shown that when individuals cannot establish effective social connections with colleagues in their daily work, their sense of belonging and emotional attachment to the organization decline significantly (Wright et al., 2006; Lam and Lau, 2012), a finding verified in multiple empirical investigations (Uysal and Kılıçkaya, 2023). For railway police officers, higher OI is associated with stronger JE (Tang et al., 2018). Yet when WL steadily erodes their OI, the psychological bond between the individual and the organization is severed. The perceived alignment between their sense of mission and organizational goals weakens, as does their willingness to make sacrifices—such as forfeiting professional identity and emotional ties upon leaving the organization. Accordingly, the following hypothesis is proposed:

*H3*: OI mediates the relationship between WL and JE.

More critically, RS and OI are not independent mediating pathways; a serial transmission relationship may exist between them. Social identity theory holds that confirmation of ingroup membership is a prerequisite for individuals to develop OI. Only when individuals can clearly perceive their role position within the group and confirm that they meet the expectations of the group prototype will the depersonalization effect occur—that is, internalizing the organization’s values and norms as part of the self-concept (Turner, 1985; Hogg and Terry, 2000). What RS signals, however, is precisely the loss of this prerequisite. Role ambiguity prevents individuals from clearly defining what kind of person the organization expects them to be. Role conflict leaves individuals torn among multiple identity demands. Role overload keeps individuals in a state of passive coping, eroding their sense of control over work. Together, these three forces fundamentally undermine the cognitive foundation of OI. A member who cannot even accurately position their own role will find it difficult to integrate organizational identity into their self-concept. Prior research has confirmed the negative predictive effect of RS on OI (Bright, 2022; Wu, 2019). Notably, this erosion may not be a one-time static event but rather a cumulative process: WL exposes individuals to a prolonged state of ambiguous self-categorization, RS steadily accumulates as a result, and OI is gradually worn away within this accumulation. This suggests that the impact of WL on JE may operate not only through the separate pathways of RS or OI but along a potential serial transmission chain. Accordingly, the following hypothesis is proposed:

More critically, RS and OI are not independent mediating pathways; a serial transmission relationship may exist between them. Social identity theory holds that confirmation of ingroup membership is a prerequisite for individuals to develop OI. Only when individuals can clearly perceive their role position within the group and confirm that they meet the expectations of the group prototype will the depersonalization effect occur—that is, internalizing the organization‘s values and norms as part of the self-concept (Turner, 1985; Hogg and Terry, 2000). What RS signals, however, is precisely the loss of this prerequisite. Role ambiguity prevents individuals from clearly defining what kind of person the organization expects them to be. Role conflict leaves individuals torn among multiple identity demands. Role overload keeps individuals in a state of passive coping, eroding their sense of control over work. Together, these three forces fundamentally undermine the cognitive foundation of OI. From the perspective of self-categorization theory, OI is not a given state but an outcome that depends on individuals’ ability to achieve a clear and stable self-categorization within the group (Turner, 1985). This self-categorization process requires that individuals first understand the group‘s norms, expectations, and their own position relative to these standards—in other words, a clear role perception is the cognitive prerequisite for identity formation. RS, particularly role ambiguity, directly obstructs this initial self-categorization process by depriving individuals of the information necessary to define their “self-in-group” prototype. Role conflict further compounds this by presenting incompatible behavioral expectations, which undermines the perceived legitimacy and desirability of group membership. Role overload consumes the limited cognitive resources needed to engage in the reflective processes that sustain identification (Ashforth and Mael, 1989). Thus, RS is conceptually and temporally antecedent to OI, because the cognitive clarity of one’s role is a prerequisite for emotional attachment to the group. Empirical research has provided support for this temporal ordering. For instance, a study examining RSors among police officers found that role ambiguity had a negative and significant effect on OI, while role conflict also negatively affected OI (Kanbur and Canbek, 2017). Similarly, integrating role theory and OI literature, researchers have demonstrated that role conflict undermines employees’ adaptive performance through its damaging effect on OI (Patil and Tewfik, 2016). Within the Chinese policing context, research has further confirmed that job stress is negatively associated with OI, and that this relationship holds significant practical implications for police force management (Lu et al., 2015). A member who cannot even accurately position their own role will find it difficult to integrate organizational identity into their self-concept. Accordingly, the following hypothesis is proposed:

*H4*: RS and OI form a serial mediation pathway between WL and JE.

### 2.5 PSM

PSM refers to an individual’s predisposition to serve the public interest. It encompasses attraction to participation in public affairs, commitment to public values, compassion, and a spirit of self-sacrifice (Perry and Wise, 1990). Research on this construct spans a wide range of populations, including police officers (Liu et al., 2015), civil servants (Giauque et al., 2012), teachers (Andersen et al., 2014), medical personnel (Jensen et al., 2019), and non-profit organization employees (Word and Carpenter, 2013). Studies on police have shown that PSM serves as a vital psychological resource that drives officers to remain at their posts and fulfill their duties with dedication. Officers with high PSM demonstrate stronger work engagement and organizational commitment (Lin, 2017).

However, the impact of PSM on employee well-being is not uniformly positive. In recent years, scholars have begun to examine its adverse effects in resource-depleted contexts. Research has found that when individuals with high PSM work in environments lacking organizational support, the gap between their elevated occupational expectations and the reality of their working conditions can trigger more severe job burnout and psychological withdrawal (Schott and Ritz, 2018; Giauque et al., 2012). Similarly, an empirical study on frontline police officers confirmed that in adverse situations marked by insufficient organizational resources and high hindrance job demands, PSM significantly amplified the positive effect of work stress on job burnout (Sha and Deng, 2024).

**Figure 1:**
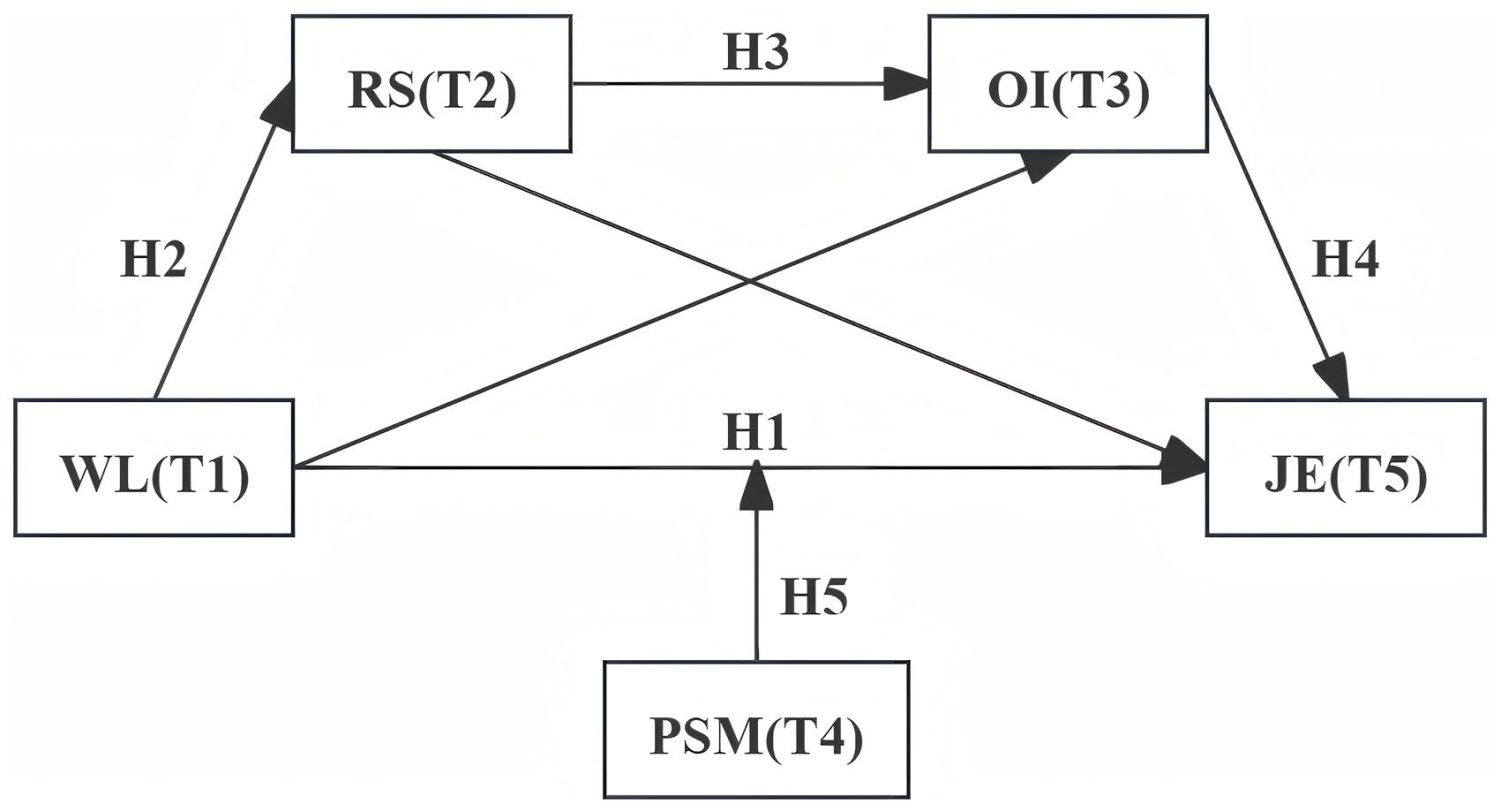
Hypothesized longitudinal moderated serial mediation model.

Whether an individual perceives fairness depends on their judgment of the ratio between their inputs and the rewards they receive. When an individual perceives their inputs to substantially outweigh the rewards obtained, a sense of inequity emerges, which may lead to coping responses such as reducing inputs or psychologically withdrawing (Adams, 1965). In this context, “inputs” include not only working hours and physical exertion but also contributions at the emotional, belief, and value levels. “Rewards” encompass not only material compensation but also the recognition, support, and sense of belonging provided by the organization (Sun and Huang, 2004). For railway police officers with high PSM, the sense of mission to serve the public and uphold justice constitutes a core spiritual input in their self-concept. From the perspective of social identity theory, these officers have internalized their professional mission as a defining feature of their organizational identity—they do not merely work for the organization; they derive a fundamental sense of who they are from their organizational membership (Tajfel and Turner, 1979). When WL deprives them of the social interaction and peer feedback necessary to sustain this identity, the very foundation of their organizational self-concept is threatened. From the lens of equity theory, this threat translates into a severe perceived imbalance: high-PSM officers invest not only time and physical energy but also ideals, beliefs, and a willingness to dedicate themselves to their work, yet WL signals a profound deficit in organizational rewards—namely, social support, team belonging, and value recognition. Thus, the identity-based expectations of high-PSM officers, when unmet, generate a more acute sense of inequity than that experienced by their low-PSM counterparts. The former bring stronger mission and dedication to their work, yet they are equally confronted with—and perhaps more sensitive to—interpersonal alienation and a lack of value recognition. This severe input-reward imbalance may trigger a strong sense of inequity, prompting individuals to reduce their inputs or psychologically withdraw, which in turn manifests as a decline in organizational attachment. Building on this logic, the following hypothesis is proposed:

*H5*: PSM moderates the relationship between WL and JE (Figure 1).

## 3 Participants and methods

### 3.1 Participants and procedure

This study adopted a cluster sampling method to select participants. Because a complete personnel list of railway police officers is protected by confidentiality regulations and could not be obtained through open channels, railway police detachments were used as the sampling unit. Specifically, several detachments under the Beijing and Shanghai Railway Public Security Bureaus served as the primary sampling clusters, while detachments from other bureaus—including Shenyang, Wuhan, and Jinan—were also included to increase the geographic diversity of the sample and strengthen the external validity of the findings. Nevertheless, this sampling strategy is not without limitations. The primary reliance on detachments from the Beijing and Shanghai bureaus may have introduced selection bias, as these regions represent economically developed, high-density railway networks with comparatively greater resource allocation. This geographical concentration could overrepresent officers from urban, resource-rich contexts and underrepresent those from less developed, remote, or resource-scarce regions where railway policing conditions may differ substantially. Therefore, the present sample may not fully capture the diversity of the railway police population across China, and caution is warranted when generalizing the findings to other railway policing systems.

Data were collected using a five-wave longitudinal design conducted from March to July 2025, with a one-month interval between waves and a total follow-up period of 4 months. The one-month interval between waves was chosen based on two considerations. First, a one-month interval is sufficiently long to capture meaningful changes in the psychological constructs under investigation (e.g., the accumulation of RS and the gradual erosion of OI), while being short enough to minimize the risk of historical events or extraneous factors that could confound the observed relationships. Second, this interval balances the need for temporal separation of measurements—to reduce common method bias and establish temporal precedence—with the practical feasibility of maintaining participant retention. If observations are spaced too far apart, the resulting data can support misleading conclusions, whereas spacing observations relatively close together provides a more veridical picture of the process of interest (Collins and Graham, 2002). In fact, optimal time lags in panel studies are usually quite short, which calls for more “shortitudinal” research in the literature (Dormann and Griffin, 2015). A longitudinal study on perceived work stress among Chinese employees conducted over the span of 1 month has demonstrated the feasibility and appropriateness of this design (Cai et al., 2025). The measurement content at each wave was as follows: T1 assessed WL and demographic variables, including gender, duty mode, age, and years of police service; T2 assessed RS; T3 assessed OI; T4 assessed PSM; and T5 assessed JE. The number of items per wave ranged from 11 to 14, with a completion time of approximately three to 5 min, so as to minimize participant burden. All questionnaires were created on the Tencent Questionnaire platform and distributed as links via internal liaisons in each detachment through work group messages. Prior to each wave, the liaisons explained the purpose of the study, the voluntary nature of participation, and the anonymization procedures. Participants completed the questionnaires only after reading the informed consent form and agreeing to take part. To enable cross-wave matching while preserving anonymity, each questionnaire required participants to enter a unique code consisting of the last four digits of their mobile phone number combined with their month of birth. This code contained no personally identifiable information and was used solely for matching purposes during data analysis.

**Figure 2:**
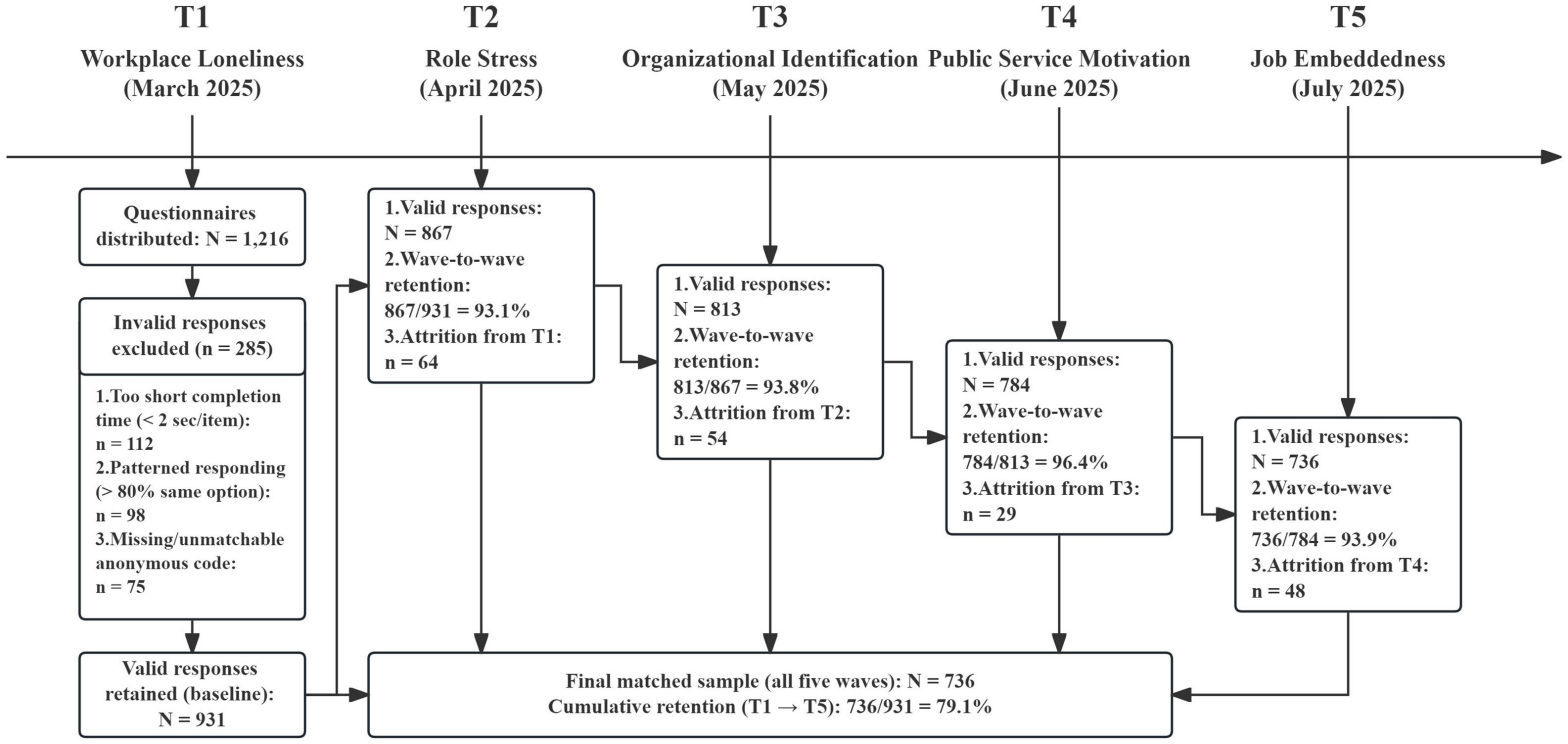
Participant flow diagram.

To reduce sample attrition across waves, several measures were implemented. Detachment liaisons sent reminders on the third and sixth days after each wave was distributed. Each wave remained open for 7 days; failure to respond within this window was treated as a missing response for that wave. At T1, participants were informed that those who completed all five waves would receive souvenirs from the People’s Public Security University of China. After each wave, data were screened according to the following criteria: excessively short completion time (an average of less than 2 sec per item), patterned responding (selecting the same option for more than 80% of items), and missing or unmatchable anonymous codes. A total of 1,216 questionnaires were distributed at T1, yielding 931 valid responses. At T2, 867 valid responses were collected (retention rate: 93.1%); at T3, 813 (retention rate: 93.8%); at T4, 784 (retention rate: 96.4%); and at T5, 736 (retention rate: 93.9%). The final matched sample across all five waves comprised 736 officers, with a cumulative retention rate of 79.1%. Of the valid sample, 657 were male (89.27%) and 79 were female (10.73%). The mean age was 33.18 years (SD = 11.46), and the mean years of police service was 6.11 (SD = 6.36). The single-officer patrol model was the predominant duty mode, accounting for 589 officers (80.03%). A participant flow diagram summarizing questionnaire distribution, response validity screening, and sample retention across the five waves is presented in Figure 2.

### 3.2 Methods

All scales in this study utilized a 5-point Likert scoring method, ranging from 1 (completely disagree) to 5 (completely agree). Higher total scores represent a higher degree of agreement with the statements.

#### 3.2.1 WL scale

The WL Scale developed by Wright et al. (2006) and revised by Mao (2013) was used. Drawing on the contextualized approach of Chung et al. (2022) for the Korean police population, adaptive adjustments were made to the scale’s phrasing for the railway police work context. The scale includes two dimensions: existential loneliness and interpersonal loneliness, totaling 10 items. In this study, the scale’s Cronbach’s *α* coefficient was 0.928, and McDonald’s omega (*ω*) coefficient was 0.934.

#### 3.2.2 RS scale

The measurement of RS was based on the original scale by Peterson et al. (1995), translated and revised by Li and Zhang (2009). It integrates three sub-dimensions: role ambiguity, role conflict, and role overload, totaling 11 items. This tool has accumulated good reliability and validity evidence among Chinese special police populations (Xiao et al., 2023). This study yielded a Cronbach’s α coefficient of 0.881 and a McDonald’s omega (ω) coefficient of 0.886.

#### 3.2.3 OI scale

The evaluation of OI utilized the classic unidimensional scale developed by Mael and Ashforth (1992), consisting of 6 items. The applicability of this scale in the context of Chinese police organizations has received empirical support (Zhao, 2021). In this study, the Cronbach’s α coefficient was 0.919, and the McDonald’s omega (ω) coefficient was 0.920.

#### 3.2.4 PSM scale

The measurement tool for PSM integrated the prototype scale by Kim et al. (2013) with the Chinese revised version by Bao and Li (2016). Adaptive adjustments were made for the railway police occupational context based on the contextual adaptation experience of Lin (2017) for police personnel, combined with literature review and frontline interviews. The scale covers four dimensions: attraction to policy-making, commitment to the public interest, compassion, and self-sacrifice, totaling 8 items. The Cronbach’s α coefficient in this study was 0.858, and McDonald’s omega (ω) coefficient was 0.873.

#### 3.2.5 JE scale

The JE Scale developed by Crossley et al. (2007) was used, with adaptive modifications based on the revised Chinese context version by Yang et al. (2019). The scale is unidimensional, with 11 items. The Cronbach’s α coefficient was 0.912, and McDonald’s omega (ω) coefficient was 0.915.

#### 3.2.6 Control variables

Gender, duty mode, age, and years of police service were included as control variables, all measured at T1. Gender was coded as a dichotomous variable (male = 0, female = 1), duty mode was treated as a multinomial categorical variable, and age and years of police service were treated as continuous variables. These variables were statistically controlled in subsequent tests of direct effects, serial mediation, and moderation effects. The selection of control variables was guided by theoretical and empirical considerations. Age and gender were included as demographic characteristics have been routinely controlled in WL and JE research, although findings regarding their effects have been mixed, with WL not significantly associated with age or gender (Brady et al., 2022) yet age being identified as a significant predictor of loneliness in some contexts (Firoz et al., 2022). Duty mode was controlled because single-officer patrol methods limit officers‘opportunities for social interaction, and police work itself has been found to lead to social isolation (Skolnick, 2005). Years of police service was included as tenure has been shown to predict JE (Gunnólfsdóttir, 2016), and long-term workers report stronger identification with their organization than short-term workers (Barker and Tompkins, 1994). By statistically controlling for these variables, we aimed to reduce the risk of spurious associations and isolate the unique effects of the core psychological constructs.

#### 3.2.7 Scale adaptation and validation

Four of the five scales employed in this study—WL, RS, PSM, and JE—underwent contextual adaptation for the railway police population. The adaptation process followed established guidelines for cross-cultural and contextual instrument adaptation, comprising three sequential steps.

Step 1: Contextual wording revision. Based on a comprehensive review of the railway police literature and frontline interviews with five active-duty railway police officers, we identified occupation-specific terms and scenarios that were incorporated into the scale items. For example, items referring to generic “colleagues” or “workplace” were revised to specify “fellow railway police officers” and “on-train duty contexts” to enhance contextual relevance. This step ensured semantic equivalence—that the meaning of each item was accurately conveyed within the railway police context.

Step 2: Expert review. The adapted versions were reviewed by a panel of three experts in occupational health psychology and two senior railway police administrators to assess content validity. Each expert evaluated the relevance and clarity of each adapted item on a 4-point scale. Items with a content validity index (CVI) below 0.80 were revised or eliminated. The final adapted scales achieved a CVI of 0.85 or above for all scales.

Step 3: Pilot testing. A pilot study was conducted with 50 railway police officers (not included in the main sample) to assess the construct validity of the adapted scales. Confirmatory factor analyses (CFA) were performed for each scale to verify that the adapted versions retained the factor structures of the original instruments. The results showed acceptable model fit for all adapted scales: WL (CFI = 0.92, RMSEA = 0.06), RS (CFI = 0.91, RMSEA = 0.07), PSM (CFI = 0.90, RMSEA = 0.06), and JE (CFI = 0.93, RMSEA = 0.05). These results provide preliminary evidence for the construct validity of the adapted scales in the railway police population.

### 3.3 Statistical processing

After excluding invalid questionnaires, attrition analysis was first conducted between the five-wave matched sample and the participants who dropped out after completing only T1. Chi-square tests and independent-samples t-tests were used to compare differences between the two groups in gender, age, years of police service, duty mode, and T1 WL scores, in order to assess the randomness of sample attrition. Subsequently, common method bias testing, descriptive statistics, and correlation analysis were performed using SPSS 28.0. The serial mediation model was tested using Model 6 in the PROCESS macro, and the significance of indirect effects was evaluated using the bootstrap method with 5,000 resamples. Moderation effects were tested using hierarchical regression analysis.

## 4 Results

### 4.1 Attrition analysis

To examine whether sample attrition was random, completers—participants who completed all five waves (*N* = 736)—were compared with dropouts—participants who withdrew after T1 (*N* = 195)—on baseline characteristics, including gender, age, years of police service, duty mode, and T1 WL. As presented in Table 1, no significant differences were found between the two groups on any baseline variable (all *ps* > 0.05): gender (*χ^2^* = 0.08, *p* = 0.777), age (*t* = −0.42, *p* = 0.674), years of police service (*t* = −0.58, *p* = 0.562), duty mode (*χ^2^* = 0.28, *p* = 0.597), and T1 WL (*t* = −0.33, *p* = 0.742). These results suggest that attrition occurred largely at random and that the final matched sample (*N* = 736) remains representative of the initial T1 sample with respect to the measured baseline characteristics.

**Table 1:** Attrition analysis: Comparison between completers and dropouts on baseline variables.

Variable	Completers (*N* = 736)	Dropouts (*N* = 195)	*χ*^2^/*t*	*p*
Gender (% male)	89.27%	88.82%	0.08	0.777
Age (M ± SD)	33.18 ± 11.46	33.58 ± 11.71	−0.42	0.674
Years of police Service (M ± SD)	6.11 ± 6.36	6.39 ± 6.52	−0.58	0.562
Duty Mode (% single)	80.03%	81.58%	0.28	0.597
WL at T1 (M ± SD)	2.059 ± 0.967	2.085 ± 0.971	−0.33	0.742

WL, Workplace Loneliness. Completers, Participants who completed all five waves; Dropouts, Participants who only completed T1.

For missing data within the five-wave matched sample, listwise deletion was employed, as the proportion of missingness across waves was minimal (retention rates ranged from 93.1 to 96.4% across T2 to T5). Given the low overall attrition rate (cumulative retention rate = 79.1%) and the random pattern of attrition suggested by the above analyses, listwise deletion was considered an appropriate and conservative approach that does not substantially bias parameter estimates. Nevertheless, we acknowledge that future research may benefit from employing more sophisticated techniques such as multiple imputation to further robustify the findings.

### 4.2 Common method bias test

To test for common method bias, Harman’s single-factor test was first conducted. The results showed that a total of seven factors had eigenvalues greater than one, and the first factor accounted for only 26.435% of the total variance (< 40%), indicating that no severe common method bias was present in this study. In addition, given the inherent limitations of Harman’s single-factor test, an unmeasured latent method factor approach was applied as a further check. Specifically, we added a common method factor to the measurement model, allowing all items to load on both their respective trait factors and the common method factor. Model fit was compared between the baseline model (without the method factor) and the method factor model (with the method factor). The results showed that adding the common method factor did not significantly improve model fit: ΔCFI = 0.003, ΔTLI = 0.002, ΔRMSEA = 0.002, ΔRMR = 0.009. All of these changes were well below the conventional thresholds that would indicate serious common method bias (i.e., ΔCFI < 0.01, ΔTLI < 0.01, ΔRMSEA < 0.01) (Wen et al., 2018). These results further confirm that common method bias does not pose a significant threat to the validity of our findings.

### 4.3 Descriptive statistics and correlation analysis

Means, standard deviations, and Pearson correlations among the study variables were computed and are presented in Table 2. The correlations among variables were in the expected directions. Specifically, T1 WL was significantly and negatively correlated with T5 JE (*p* < 0.01), indicating that stronger perceptions of loneliness were associated with lower levels of JE, thereby providing preliminary support for H1. In addition, age, years of police service, and duty mode were all significantly correlated with JE (*ps* < 0.01), suggesting that these demographic variables may exert confounding effects on the core variables. They were therefore controlled as covariates in all subsequent analyses (Table 3).

**Table 2:** Means, standard deviations, and correlation matrices of the main variables (*N* = 736).

Variable	M	SD	1	2	3	4	5	6	7	8	9
1. DM	—	—	—								
2. Gender	0.107	0.309	−0.163**	—							
3. Age	33.18	11.46	−0.006	0.193**	—						
4. YS	6.11	6.36	0.045	0.207**	0.831**	—					
5. WL(T1)	2.059	0.967	−0.138**	0.041	0.118**	0.078*	—				
6. RS(T2)	2.484	0.852	−0.049	0.017	0.128**	0.104**	0.722**	—			
7. OI(T3)	3.887	0.989	0.123**	0.028	−0.012	−0.015	−0.459**	−0.417**	—		
8. JE(T5)	3.211	0.953	0.104**	−0.060	−0.178**	−0.146**	−0.621**	−0.607**	0.602**	—	
9. PSM(T4)	4.293	0.675	0.051	−0.031	−0.093*	−0.074*	−0.337**	−0.335**	0.580**	0.435**	—

DM, Duty Mode; YS, Year of police Service; WL(T1), Workplace Loneliness at Time 1; RS(T2), Role Stress at Time 2; OI(T3), Organizational Identification at Time 3; PSM(T4), Public Service Motivation at Time 4; JE(T5), Job Embeddedness at Time 5.*p < 0.05; **p < 0.01; ***p 0.001.

**Table 3:** Bootstrap analysis of longitudinal serial mediation effects (*N* = 736).

Path of influence	Corresponding hypothesis	Effect	SE	LLCI	ULCI	Proportion
WL(T1) → JE(T5)	H1	−0.244	0.037	−0.316	−0.172	40.9
WL(T1) → RS(T2) → JE(T5)	H2	−0.182	0.031	−0.243	−0.120	30.5
WL(T1) → OI(T3) → JE(T5)	H3	−0.120	0.023	−0.166	−0.078	20.1
WL(T1) → RS(T2) → OI(T3) → JE(T5)	H4	−0.051	0.015	−0.081	−0.023	8.5
Total effect	-—	−0.597	0.029	−0.654	−0.541	100

LLCI refers to the lower limit of the 95% Bootstrap confidence interval, and ULCI refers to the upper limit of the 95% Bootstrap confidence interval. T1-T5 indicate the measurement wave for each variable.

### 4.4 Mediation effect test of RS and OI

**Figure 3:**
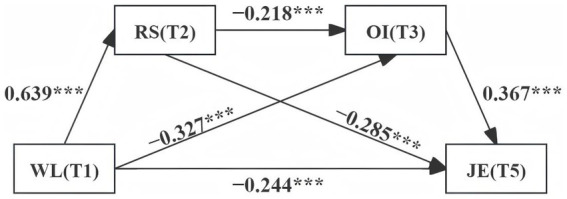
Path coefficients of the longitudinal serial mediation model.

All variables were standardized prior to analysis. T1 WL was entered as the independent variable, T5 JE as the dependent variable, and T2 RS and T3 OI as serial mediators. Gender, duty mode, age, and years of police service were included as control variables. The results (see Figure 3) showed that T1 WL significantly and positively predicted T2 RS [*β* = 0.639, *t* = 27.997, *p* < 0.01, 95% CI (0.594, 0.683)] and significantly and negatively predicted T3 OI [*β* = −0.327, *t* = −6.743, *p* < 0.01, 95% CI (−0.422, −0.232)]. T2 RS significantly and negatively predicted T3 OI [*β* = −0.218, *t* = −3.996, *p* < 0.01, 95% CI (−0.326, −0.111)] and T5 JE [*β* = −0.285, *t* = −7.040, *p* < 0.01, 95% CI (−0.364, −0.205)]. T3 OI significantly and positively predicted T5 JE [*β* = 0.367, *t* = 13.518, *p* < 0.01, 95% CI (0.314, 0.420)]. The direct effect of T1 WL on T5 JE was significant [*β* = −0.244, *t* = −6.662, *p* < 0.01, 95% CI (−0.316, −0.172)], as was the total effect [*β* = −0.597, *t* = −20.739, *p* < 0.01, 95% CI (−0.654, −0.541)]. These results indicate that T2 RS and T3 OI play a significant partial serial mediating role between T1 WL and T5 JE.

Bootstrap analysis of the mediating pathways further showed that the total indirect effect of T1 WL on T5 JE was −0.353, accounting for 59.1% of the total effect (−0.597). The decomposition of each mediating pathway is presented in Table 4. The indirect effect via T2 RS was −0.182, explaining 30.5% of the total effect; the indirect effect via T3 OI was −0.120, accounting for 20.1% of the total effect; and the serial mediation pathway through both mediators yielded an effect of −0.051, accounting for 8.5% of the total effect. None of the 95% confidence intervals for these indirect effects included zero, indicating that the mediation through RS, OI, and their serial combination were all statistically significant. These findings demonstrate that T1 WL not only directly reduces T5 JE among railway police officers, but also exerts its negative influence indirectly by first heightening T2 RS, which in turn erodes T3 OI. Thus, Hypotheses H1 through H4 were supported.

**Table 4:** Hierarchical regression analysis of the moderating effect of public service motivation (*N* = 736).

Variable	Model 1		Model 2		Model 3	
	*β*	*t*	*β*	*t*	*β*	*t*
Gender	−0.01	−0.347	−0.009	−0.312	−0.006	−0.228
DM	0.02	0.669	0.018	0.655	0.02	0.743
Age	−0.073	−1.407	−0.062	−1.254	−0.093	−1.927
YS	−0.037	−0.709	−0.034	−0.691	0.006	0.129
WL(T1)	−0.606	−20.739**	−0.524	−17.759**	−0.509	−17.693**
PSM(T4)			0.249	8.523**	0.287	9.915**
Int_1					−0.181	−6.586**
*R^2^*	0.397		0.452		0.483	
Δ*R^2^*	0.397		0.055		0.031	
*F*	96.255**		100.192**		97.068**	
Δ*F*	96.255**		72.645**		43.376**	

DM, Duty Mode; YS, Year of police Service; WL(T1), Workplace Loneliness at Time 1; PSM(T4), Public Service Motivation at Time 4; Int_1 = WL(T1) × PSM(T4). *p < 0.05; **p < 0.01; ***p 0.001.

### 4.5 Moderation effect test of PSM

Hierarchical regression analysis was conducted to examine the moderating effect of T4 PSM on the relationship between T1 WL and T5 JE (see Table 4). All continuous variables were standardized prior to analysis, and the interaction term was generated as the product of standardized T1 WL and standardized T4 PSM. Gender, duty mode, age, and years of police service were included as control variables, while T5 JE was left untransformed as the dependent variable.

Model 1 included the control variables and T1 WL. Model 2 added T4 PSM. Model 3 further added the interaction term between T1 WL and T4 PSM. As shown in see Table 4, T1 WL significantly and negatively predicted T5 JE in Model 1 (*β* = −0.606, *p* < 0.01). After T4 PSM was entered in Model 2, it significantly and positively predicted T5 JE (*β* = 0.249, *p* < 0.01), while the negative effect of T1 WL was somewhat attenuated but remained significant (*β* = −0.524, *p* < 0.01). When the interaction term was added in Model 3, it reached significance (*β* = −0.181, *p* < 0.01), and the model explained significantly more variance than Model 2 (Δ*R^2^* = 0.031, Δ*F* significant). The negative coefficient of the interaction term indicates that the negative predictive effect of T1 WL on T5 JE grew stronger as T4 PSM increased. Thus, H5 was supported.

**Figure 4:**
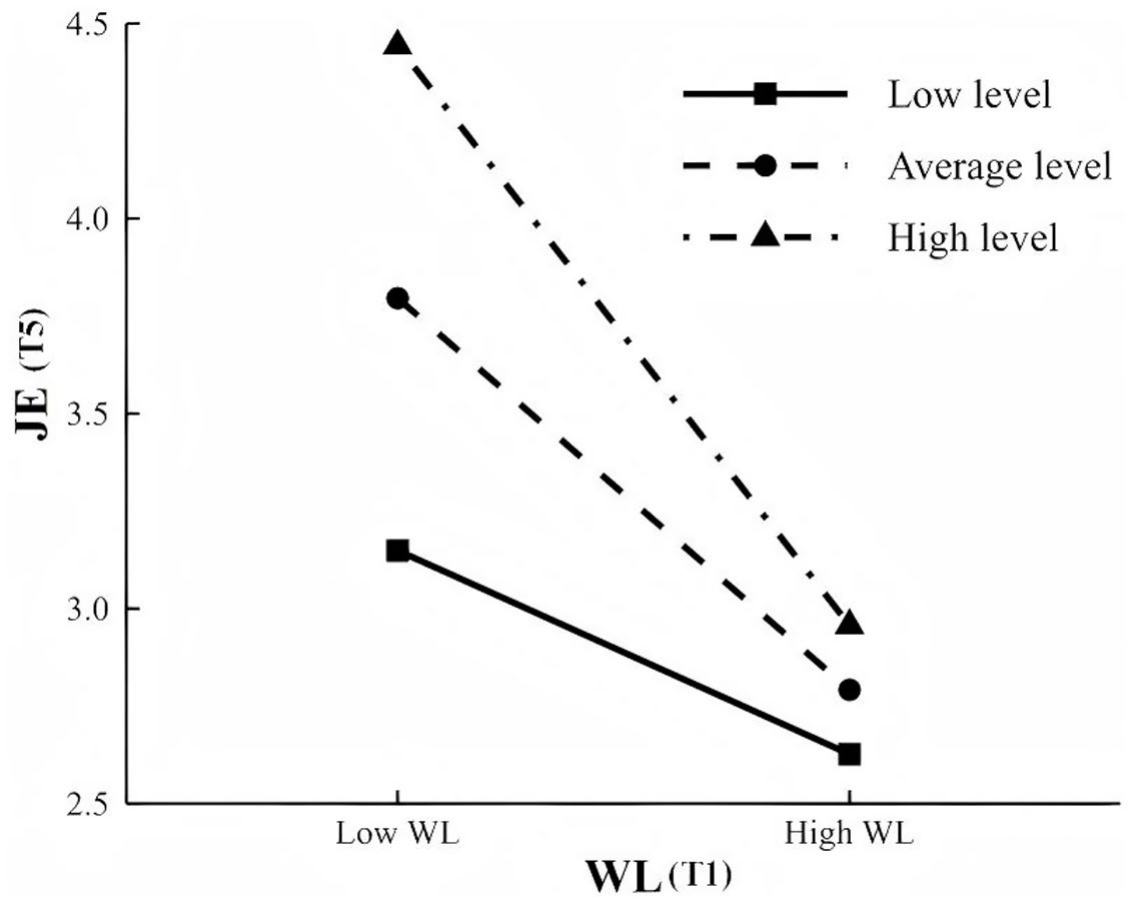
Simple slopes of the effect of T1 workplace loneliness on T5 job embeddedness at high, medium, and low levels of T4 public service motivation.

To further clarify the form of this moderation effect, simple slope analysis was conducted. As shown in Figure 4, at low levels of T4 PSM (−1 SD), the negative effect of T1 WL on T5 JE was relatively weak [*β* = −0.339, *t* = −8.672, *p* < 0.01, 95% CI (−0.415, −0.262)]. At mean levels of T4 PSM, the negative effect strengthened [*β* = −0.502, *t* = −17.693, *p* < 0.01, 95% CI (−0.557, −0.446)]. At high levels of T4 PSM (+1 SD), the negative effect reached its strongest magnitude [*β* = −0.664, *t* = −18.395, *p* < 0.01, 95% CI (−0.735, −0.593)]. These findings indicate that, in the present sample, T4 PSM did not buffer the detrimental impact of T1 WL on T5 JE; rather, it significantly amplified this negative relationship. Consistent with equity theory, officers with high PSM regard their sense of mission—serving the public and upholding justice—as a core personal input. WL, in contrast, signals a severe deficit in organizational rewards. This stark imbalance between input and reward triggers a strong sense of inequity, which in turn accelerates the process of psychological detachment and ultimately leads to a sharper decline in JE.

## 5 Discussion

### 5.1 The influencing mechanisms of RS and OI

This study found that T1 WL indirectly led to a significant decline in T5 JE through a serial mediation pathway—by heightening T2 RS and, in turn, weakening T3 OI. This longitudinal sequence process shows that the erosion of WL on organizational attachment is not only a one-time impact, but also a dynamic transmission process that permeates layer by layer. Previous meta-analytic work has established robust cross-sectional correlations between WL and negative work outcomes, including turnover intentions, reduced commitment, and burnout (Bryan et al., 2023). However, the causal ordering of these associations has remained unclear, prompting calls for longitudinal designs to disentangle the underlying mechanisms. The present findings respond to this call by demonstrating that WL first disrupts role cognition, then corrodes the sense of belonging at the identity level, before finally manifesting as diminished JE at the behavioral level.

Within the framework of social identity theory, whether individuals can maintain a stable identification with their group depends heavily on the continuous availability of two critical pieces of information: the clarity of group boundaries and the confirmation of their own position within the group. For railway police officers, prolonged single-officer deployment means that both information channels are simultaneously cut off. When an officer handles various policing situations alone on a train, there are no colleagues nearby to serve as immediate referents. The officer cannot judge whether their approach aligns with the group’s typical practices, nor can they receive feedback from peers to confirm the normative appropriateness of their behavior. This structural information deprivation strips individuals of the basic conditions for confirming the positive distinctiveness of their ingroup through social comparison (Tajfel, 1982), and resonates with field observations that single-officer patrols intensify officers’ sense of isolation and weaken their collective identity (Shanghai Railway Public Security Bureau Office Research Group, 2022). The resulting role ambiguity, role conflict, and role overload converge at T2, manifesting as the accumulation of RS.

The mediating role of RS aligns with prior evidence that WL is more than an affective experience—it represents a loss of access to role-related feedback and social information (Lam and Lau, 2012), and that deficient social support in the workplace significantly heightens perceptions of RS (Chiu et al., 2017). By identifying RS as a key cognitive pathway, this study extends earlier cross-sectional findings that RS negatively predicts JE (Guan et al., 2025) and provides longitudinal evidence for the temporal precedence of RS in the loneliness–embeddedness link.

The transmission from T2 RS to T3 OI is not a simple summation of two parallel pathways but a directional, sequential process. The formation of OI depends heavily on an individual’s ability to clearly perceive their belongingness to the group—a process that Ashforth and Mael (1989) described as the integration of organizational membership into the self-concept. RS, however, obstructs this process at the cognitive level. Role ambiguity causes individuals to doubt their membership qualifications. Role conflict leaves them without a unified sense of direction among multiple courses of action. Role overload steadily depletes the psychological resources needed to maintain group bonds. The cumulative effect of these three pressures at T2 becomes evident at T3 as the erosion of OI: individuals gradually detach themselves from the group cognitively. Consistent with prior research, WL has been shown to reduce OI (Uysal and Kılıçkaya, 2023), while OI positively predicts JE (Tang et al., 2018). The present study integrates these separate strands of evidence into a coherent serial mediation model. Prior research has noted the buffering function of OI against the negative effects of RS (Kuo, 2015; Bright, 2022), but the longitudinal findings of the present study reveal the other side of this relationship—RS itself is also an important antecedent that erodes OI. This bidirectional relationship suggests that while strong identification can temporarily shield employees from the adverse effects of stress, prolonged RS can eventually wear down the very identification that once served as a buffer.

Ultimately, the weakening of T3 OI manifests at T5 as a decline in JE. When the psychological bond between the individual and the organization is severed, the emotional ties that sustain organizational attachment break down, and the individual’s intention to stay correspondingly wanes, as Mitchell et al. (2001) posited in their original conceptualization of JE as a composite of links, fit, and sacrifice. Notably, even after controlling for the indirect effects of T2 RS and T3 OI, the direct effect of T1 WL on T5 JE remained significant. This persistent direct effect suggests that the erosion of organizational attachment by communication deprivation involves mechanisms that extend beyond the pathways of role cognition and identity, possibly including the direct depletion of emotional resources or the breakdown of informal social networks within the organization—possibilities that warrant further investigation in future research.

### 5.2 The moderating role of PSM

This study found that T4 PSM did not buffer the negative impact of T1 WL on T5 JE; rather, it significantly amplified it. This means that railway police officers with stronger PSM experienced a more pronounced decline in organizational attachment in lonely work situations. This finding adds new longitudinal evidence to the growing scholarly discussion on the “dark side” of PSM. The traditional view emphasizes the positive functions of PSM as a prosocial orientation, holding that individuals with high PSM exhibit greater work engagement and organizational commitment (Liu et al., 2015). However, Giauque et al. (2012), in their study of Swiss civil servants, found that when organizational support was insufficient, PSM instead intensified declines in job satisfaction. Schott and Ritz (2018) further argued from a theoretical perspective that PSM may heighten individuals’ expectations toward their organization, making them more vulnerable to frustration when those expectations go unmet—a mechanism that becomes particularly salient in contexts of organizational resource scarcity. In the present study, T4 PSM was measured at T4, temporally independent of the T1 independent variable and the T2 and T3 mediators, which helps to rule out measurement context interference and provides more robust causal evidence for the moderation effect.

Whether an individual perceives fairness depends on the ratio between inputs and rewards (Adams, 1965), and the very definition of inputs and rewards hinges largely on the individual’s own cognitive framework and value weighting. For those with low PSM, the relationship between work and organization more closely resembles a transactional contract: individuals invest time, physical effort, and professional skills, while the organization provides pay, security, and basic working conditions. In this psychological frame, although T1 WL is a negative experience, it does not necessarily disrupt their judgment of the equity equation, because social support and value recognition do not fall within the scope of their core inputs and expected rewards. This resonates with a potential boundary condition noted in Bryan et al.'s (2023) meta-analysis—that the detrimental effects of WL may vary depending on individuals’ expectations regarding social relationships at work. The psychological structure of those with high PSM is markedly different. Perry and Wise (1990) defined PSM as an individual’s intrinsic predisposition to serve the public interest, meaning that for highly mission-driven individuals, the ideals and beliefs of serving the public and upholding justice are not merely external job demands but have been integrated as core components of their self-value system. When these officers take up their duty posts, what they invest includes not only working hours and professional skills but also the belief commitments and identity expectations built around public values. This difference in input structure directly determines the outcome of equity evaluation and also explains why highly public service-motivated individuals experience more intense psychological disequilibrium when facing the same lonely work situation. The Job Demands-Resources (JD-R) model offers a complementary theoretical lens for explaining the paradoxical moderating effect of PSM. According to the JD-R model, employees’ well-being and behavioral performance are determined by the dynamic balance between job demands and job resources (Bakker and Demerouti, 2007). PSM is typically conceptualized as a personal resource that is expected to buffer the negative effects of job demands. However, emerging evidence suggests that PSM may have a dark side, particularly in contexts where organizational resources are scarce. Specifically, PSM elevates individuals’ expectations toward their work, thereby potentially intensifying rather than alleviating public employees’ perceptions of stress (Giauque et al., 2013). Schott and Ritz (2018) similarly noted that the dark side of PSM emerges when it compounds job demands in resource-scarce environments, underscoring the critical role of organizational support. In the present study, WL can be conceptualized as a chronic emotional job demand that continuously depletes employees’ psychological energy and social relational resources. Under such circumstances, high PSM paradoxically fails to buffer—and may even fail to mitigate—the strain induced by loneliness. Indeed, PSM can produce both positive effects through challenge-related stress pathways and negative effects through hindrance-related stress pathways, with the latter being particularly pronounced when organizational resources are insufficient to support employees’ mission-driven expectations (Zhang and Liu, 2024). Consequently, when railway police officers with high PSM are confronted with intense job demands (loneliness) while simultaneously being deprived of essential organizational resources (e.g., peer support, value recognition, and organizational belonging), they undergo a more severe resource depletion process.

Highly public service-motivated individuals naturally have higher expectations of organizational rewards. Sun and Huang (2004) pointed out that employees’ expectations of organizational returns are not confined to material aspects but also include the organization’s recognition of their value and emotional support. This is precisely what those with high PSM look for—not only salary and security but also organizational acknowledgment of their mission commitment and team resonance with their belief pursuits. The reality that T1 WL represents, however, is precisely the disappointment of these expectations. The single-officer patrol model means that daily social interaction is reduced to a minimum; individuals can neither share the ethical perplexities and professional insights encountered in the law enforcement process with their peers, nor feel a sense of belonging and support through team collaboration. The Shanghai Railway Public Security Bureau Office Research Group (2022) similarly found in field investigations that the sense of isolation and weakened collective identity brought about by single-officer patrols is a prominent factor undermining team cohesion. Viewed through the lens of equity theory, the input side bears heavy belief commitments, while the reward side returns only interpersonal alienation and a lack of value recognition; the ratio between the two is thus drastically skewed.

More critically, this skew has a self-reinforcing character among those with high PSM. Belief inputs differ from material inputs: the latter can be quantified and reduced based on equity evaluations (Adams, 1965), whereas the former, once formed, have a strong tendency toward self-perpetuation—individuals are inclined to justify their initial choices through continued commitment rather than acknowledge the failure of their belief investments (Staw, 1976). This means that when faced with insufficient organizational rewards, highly PSM officers do not quickly adjust their input levels to restore equity the way those with a transactional mindset do; instead, they continuously accumulate a sense of inequity through sustained high inputs, persistently scrutinizing their fit with the organization against high standards over an extended period (Vogel and Kroll, 2016). The “calling” of public service, when it receives no organizational response, can turn from a source of motivation into a psychological burden (Giauque et al., 2012). The present study also forms cross-sample corroboration with the empirical research of Sha and Deng (2024) on frontline police officers, which similarly revealed that under conditions of insufficient organizational support, PSM strengthens the facilitating effect of stress on job burnout. It can thus be seen that cultivating PSM is not a once-and-for-all positive strategy for organizations. When organizations cannot simultaneously improve staffing levels, strengthen social support, and provide value feedback commensurate with officers’ sense of mission, highly mission-driven individuals may instead face deeper psychological difficulties precisely because of the elevated expectations embedded in their beliefs. Building an organizational ecology in which the sense of mission can take root rather than be gradually depleted is the key proposition for truly transforming PSM into workforce stability.

### 5.3 Mechanisms beyond the mediation model

Although our serial mediation model was supported, it is important to note that the direct effect of WL on JE remained substantial, accounting for 40.9% of the total effect. This persistent direct effect, even after accounting for the indirect effects of RS and OI, suggests that additional explanatory mechanisms may be at play. Future research could benefit from exploring the role of unmeasured third variables that might buffer or exacerbate the negative consequences of loneliness in the workplace.

At the individual level, psychological capital and resilience represent two promising personal resources that may moderate the negative impact of WL and RS. Psychological capital (PsyCap), comprising self-efficacy, optimism, hope, and resilience, has been found to significantly buffer the detrimental effects of WL on employee outcomes, such that the negative impact on performance behaviors is less severe for individuals with high psychological capital (Firoz and Chaudhary, 2022). Resilience, defined as an individual’s ability to adapt positively in the face of adversity, may moderate the link between RS and OI, with highly resilient employees being better equipped to maintain their organizational bonds despite experiencing heightened role pressures. Empirical evidence has confirmed that resilience serves as a buffer between work stress and burnout, mitigating the adverse effects of stress on employee well-being (Hao et al., 2015). These findings suggest that both psychological capital and resilience may serve as protective factors that interrupt the transmission from WL to organizational detachment.

At the organizational level, leadership styles represent critical contextual factors that shape how employees perceive and respond to the relational deficits signaled by loneliness. Transformational leadership, characterized by vision articulation, intellectual stimulation, and individualized consideration, has been shown to play a dynamic role in reducing WL, thereby preventing employee burnout (Kloutsiniotis et al., 2022). By providing inspirational motivation and personalized support, transformational leaders may compensate for the social and emotional deficits that lonely employees experience. Similarly, servant leadership—which emphasizes leaders’ genuine concern for subordinates’ needs and well-being—has been found to reduce WL through the enhancement of employees’ bonding social capital (Chughtai, 2026). Servant leaders’ empathic orientation and commitment to follower development may create a relational environment that offsets the isolating effects of solitary work conditions. These lines of evidence suggest that both transformational and servant leadership may alter the strength of the pathways we have identified, either by reducing the initial experience of loneliness or by reshaping employees’ interpretations of their relational environment.

Incorporating these individual-level psychological resources and organizational-level contextual factors into future research could substantially enhance the explanatory power of models examining how and when WL translates into organizational detachment.

### 5.4 Policy recommendations

The following policy recommendations are offered at two levels of evidentiary grounding. Recommendations directly supported by our empirical findings flow from the core mechanisms identified in this study—specifically, the mediating role of RS and OI, and the amplifying effect of PSM in lonely work contexts. Broader professional recommendations extend beyond the direct measures of our model and reflect supplementary considerations derived from our engagement with railway police practice and the extant literature; these are offered as complementary considerations for organizational reform rather than as direct empirical implications.

#### 5.4.1 Recommendations directly supported by empirical findings

Build an on-train collaboration network. Railway police have long undertaken train security duties alone, lacking daily interaction and behavioral reference with colleagues, which is a significant contributor to the accumulation of RS and the weakening of organizational belonging. Relying solely on psychological counseling is insufficient to resolve this issue. A more effective approach lies in embedding normalized collaboration channels into the duty mechanism, enabling officers to receive immediate peer support and organizational feedback during their shifts. This can be implemented from three specific aspects. First, establish a multi-post joint prevention mechanism on trains, integrating staff from different positions such as passenger transport, vehicle maintenance, and catering services into the security information network. This should specify the concrete tasks for each post in identifying anomalies, initial stabilization, and assisting in incident management, so that the officer is no longer an isolated individual but part of a collaborative team that can be reached at any moment (Shanghai Railway Public Security Bureau Office Research Group, 2022). Second, set up instant communication channels through mobile police terminals or a train-based collaborative platform, enabling rapid information reporting and flat command transmission between the officer, the train conductor, and the command center. This ensures officers can quickly obtain analytical support when facing complex situations rather than struggling in confusion alone. Third, dynamically deploy professional support forces on key routes and during peak times, flexibly allocating them based on passenger flow data and police situation distribution, thereby providing professional backing for frontline officers and alleviating the psychological stress induced by the long-term single-officer duty model.

Perfect the emergency support system. The finding that WL significantly predicts heightened RS underscores the importance of reducing uncertainty and enhancing officers‘sense of organizational backing during solitary law enforcement. Currently, emergency response mechanisms in many regions still suffer from bottlenecks, such as ambiguous activation conditions and insufficient dispatching authority at the grassroots level. These lead to uncertainty for officers when requesting emergency assistance, which in turn exacerbates their sense of insecurity during daily duty. To address these issues, institutional design must be used to transform emergency support from elastic mobilization into a rigid guarantee. First, clearly define the activation standards and operational procedures for emergency reinforcements. This includes specifying the procedures for requesting support, the scale of response, and the rules for the transfer of on-site command in situations such as major sudden incidents on trains or a clear insufficiency of police strength, thereby eliminating frontline officers’ concerns about requesting support. Second, unify and simplify the standardized processes for case handover, implementing electronic recording and full-process traceable management to avoid increased workload for officers arising from disputes over documentary standards or evidence specifications.

#### 5.4.2 Broader professional recommendations

Officers with strong public service beliefs typically regard serving the public and upholding justice as vital pillars of their personal values. However, the interpersonal alienation and lack of professional value feedback brought about by long-term solitary duty create a significant gap between their spiritual investment and the actual support provided by the organization. This implies that officers with a stronger sense of mission are more prone to psychological withdrawal under conditions where organizational responses are lacking. The key to resolving this contradiction lies not in weakening the advocacy of professional mission but in institutionalizing reciprocal responses to officers’ spiritual dedication (Sha and Deng, 2024). First, set differentiated evaluation weights in the performance appraisal system for those who persist in independent duty posts over the long term. Incorporate officers’ practical actions in properly handling police situations and resolving disputes and conflicts into positive feedback mechanisms, so that their perseverance in daily work receives explicit organizational recognition. Second, broaden the information access channels for frontline officers, encouraging them to establish temporary security information communication mechanisms with passengers on the premise of not violating confidentiality regulations. This not only extends the officer’s antennae for security perception but also allows them to feel supportive forces from their social environment while performing their duties. Third, establish dedicated quotas or bonus points in honor selections and career development paths for frontline single-officer duty posts that endure high stress and high loneliness. This ensures that the dedication most cherished by high-mission officers is taken seriously by the organization in an institutionalized manner, rather than merely staying as verbal advocacy. Only when structural improvements in organizational support are matched with officers’ intrinsic sense of mission can public service beliefs transform from a risk factor that exacerbates withdrawal into a positive force for maintaining workforce stability.

### 5.5 Contributions, limitations, and future prospects

The primary methodological contribution of this study lies in its adoption of a five-wave longitudinal design that temporally separated the independent variable, mediators, moderator, and dependent variable, thereby effectively circumventing the parameter estimation bias commonly inherent in cross-sectional mediation analyses (Maxwell and Cole, 2007). This design provides more robust causal and temporal evidence than cross-sectional studies for the theoretical proposition that WL erodes JE layer by layer through the serial mediation pathway of RS and OI. At the theoretical level, this study advances research on the consequences of WL from a focus on direct effects toward the revelation of a longitudinal chain-like transmission mechanism, extending the application boundary of social identity theory in explaining the psychological detachment process among high-loneliness occupational groups.

Several limitations should, however, be noted. First, the study’s reliance on cluster sampling, necessitated by the confidentiality regulations of public security authorities, may have introduced selection bias that limits the generalizability of our findings. Although we included detachments from multiple bureaus to enhance geographic diversity, the sample was predominantly drawn from the Beijing and Shanghai railway police bureaus, which operate within economically developed, high-resource environments. This geographical concentration may overrepresent officers from well-resourced, urban contexts and underrepresent those from remote or less-developed regions. Such sampling constraints may affect the external validity of the serial mediation and moderation effects observed, as the psychological experiences of WL and the availability of organizational resources could differ meaningfully across regional policing contexts. This potential selection bias is compounded by the fact that our retention rate, while acceptable, may reflect differential attrition that further constrains the representativeness of the final sample. Future research should, where conditions permit, seek to establish collaborations with a broader and more systematically selected range of railway police agencies—for instance, through stratified cluster sampling that accounts for regional economic, geographic, and operational variations—to robustly test the applicability of the present model across diverse railway policing systems. Second, although the longitudinal design and statistical tests (Harman’s single-factor test and ULMC) suggest that common method bias does not pose a serious threat to the validity of our findings, a potential concern remains regarding the exclusive reliance on self-report measures. The self-report methodology may be susceptible to social desirability bias, affective influences on item endorsement, and same-source inflation of the relationships among variables. For instance, Police officers with high levels of loneliness are predisposed to forming negative perceptions of their work environment, including role expectations and organizational support. Lonely individuals allocate greater attention to threatening stimuli, tend to make negative attributions, and hold more negative evaluations of themselves and others(Spithoven et al., 2017). Accordingly, police officers experiencing loneliness may systematically interpret workplace cues from a negative perspective, thereby potentially amplifying the magnitude of the observed associations between variables in the present study. While the temporal separation of five-wave measurements in the present study partly mitigates this concern, unmeasured individual dispositions such as negative affectivity (NA) are among the primary sources of rater-induced method bias (Podsakoff et al., 2003), which may systematically influence police officers’ responses to self-report measures across all five measurement waves. Therefore, we acknowledge this limitation and encourage future investigations to incorporate multi-source data, such as supervisor ratings of job performance, peer assessments of OI, or objective indicators of turnover and absenteeism, to validate and extend our findings, thereby attenuating the potential limitations associated with self-report data. Third, the sample was drawn entirely from the railway police population, and caution is warranted when generalizing the conclusions to other high-loneliness occupational groups. Fourth, because the core variables were measured entirely separately across time points, it was not possible to construct multi-variable profiles at a single time point to identify heterogeneous subgroups. Future research could measure RS and OI simultaneously at the same time point and adopt latent transition analysis to track individuals’ dynamic shifts across different profiles.

## 6 Conclusion

Integrating Social Identity Theory and Equity Theory, this study employed a five-wave longitudinal design to examine the mechanism through which WL influences JE among Chinese railway police officers. Our findings support a serial psychological transmission pathway: WL was positively associated with elevated RS, which in turn predicted lower OI, and these sequential changes were ultimately associated with reduced JE. Specifically, the total indirect effect via RS, OI, and their serial combination accounted for 59.1% of the total effect. Notably, we found that PSM—while generally considered a positive force—paradoxically amplified the negative association between loneliness and embeddedness, such that this negative association was stronger for officers with higher levels of PSM.

These findings provide longitudinal evidence for the erosion pathway in the WL literature, extend the application of Social Identity Theory and Equity Theory to high-loneliness occupational groups, and carry practical implications for risk identification and intervention design. However, given the observational nature of our data and the specific sample of Chinese railway police officers, causal conclusions should be drawn cautiously.

## Data Availability

The raw data supporting the conclusions of this article will be made available by the authors, without undue reservation.
